# Supplementary material for: Development and Evaluation of Matrices Composed of β-cyclodextrin and Biodegradable Polyesters in the Controlled Delivery of Pindolol
Source: Pharmaceutics. 2020 May 30;12(6):500. doi: 10.3390/pharmaceutics12060500 (PMC7355766; doi:10.3390/pharmaceutics12060500)
Supplement: Supplementary file 1 [file pharmaceutics-12-00500-s001.pdf]

pharmaceutics

Supplementary Materials: Development and Evaluation of Matrices Composed of β-cyclodextrin and Biodegradable Polyesters in the Controlled Delivery of Pindolol

Agnieszka Lis-Cieplak, Filip Charuk, Marcin Sobczak, Anna Zgadzaj, Agata Drobniewska, Łukasz Szeleszczuk and Ewa Oledzka

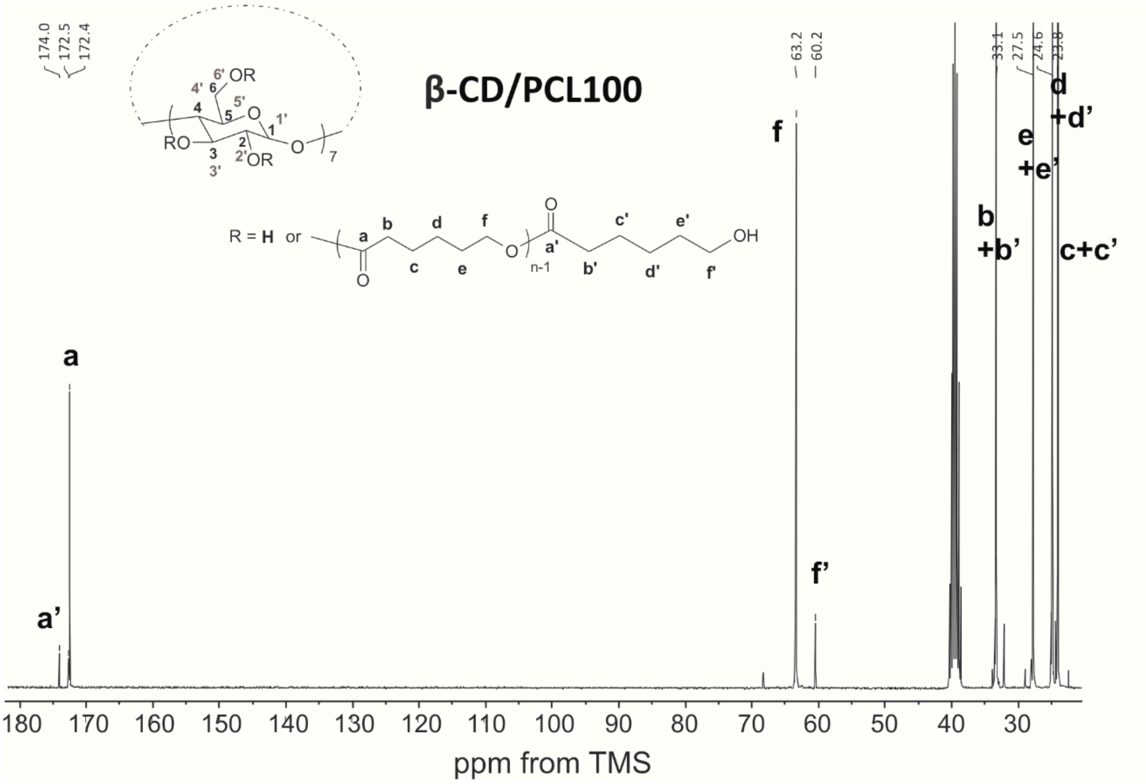

Figure S1. ^{13}C NMR spectrum of the β-CD/PCL100 polymer in DMSO-d_6.

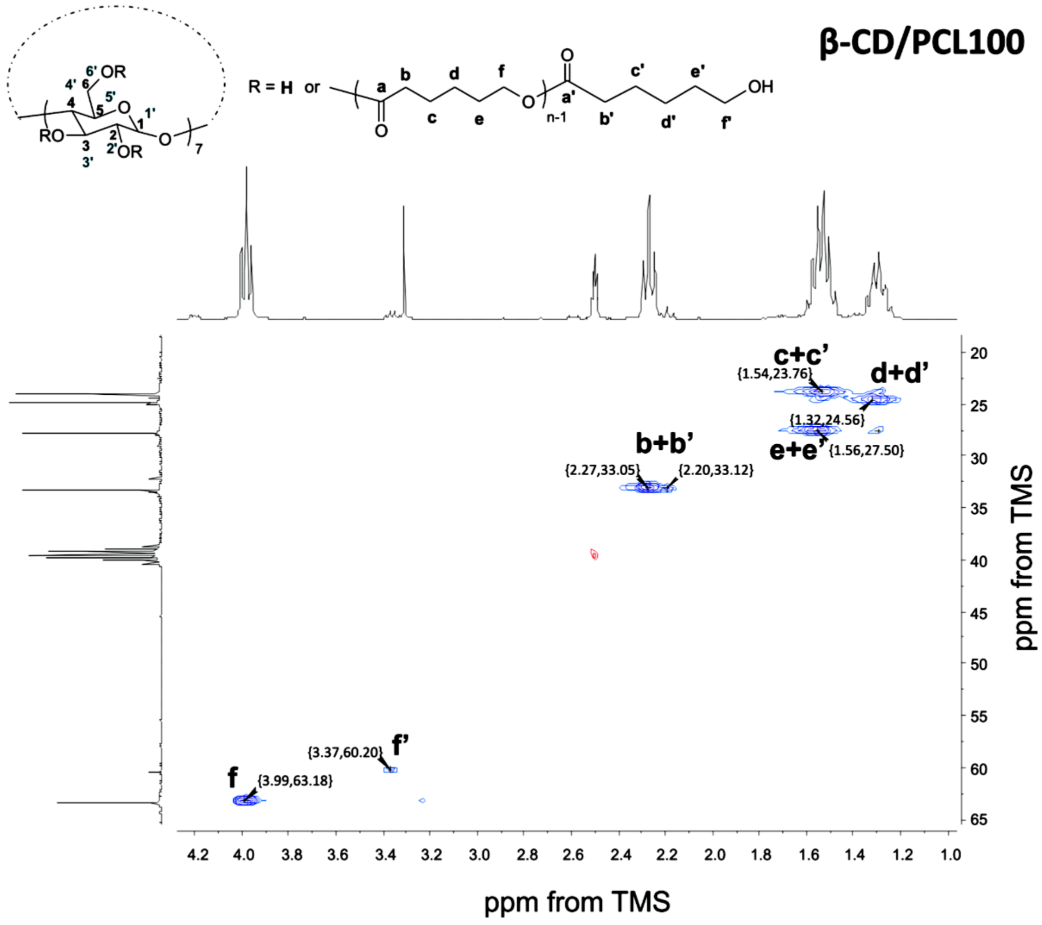

Figure S2. HSQC spectrum of the β-CD/PCL100 polymer in DMSO-d6.

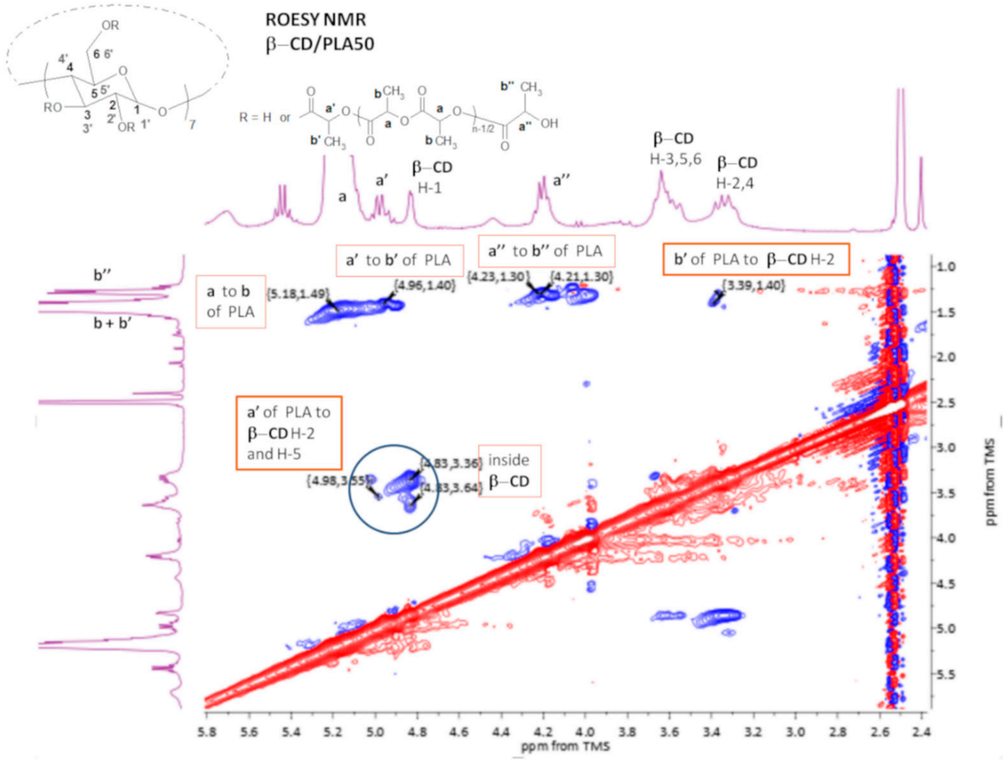

Figure S3. ROESY spectrum of the β-CD/PLA50 polymer in DMSO-d6, marked ROE cross-peaks between H-2 protons of β-CD and the first mer unit of PLA. Circled area is shown enlarged on the next figure.

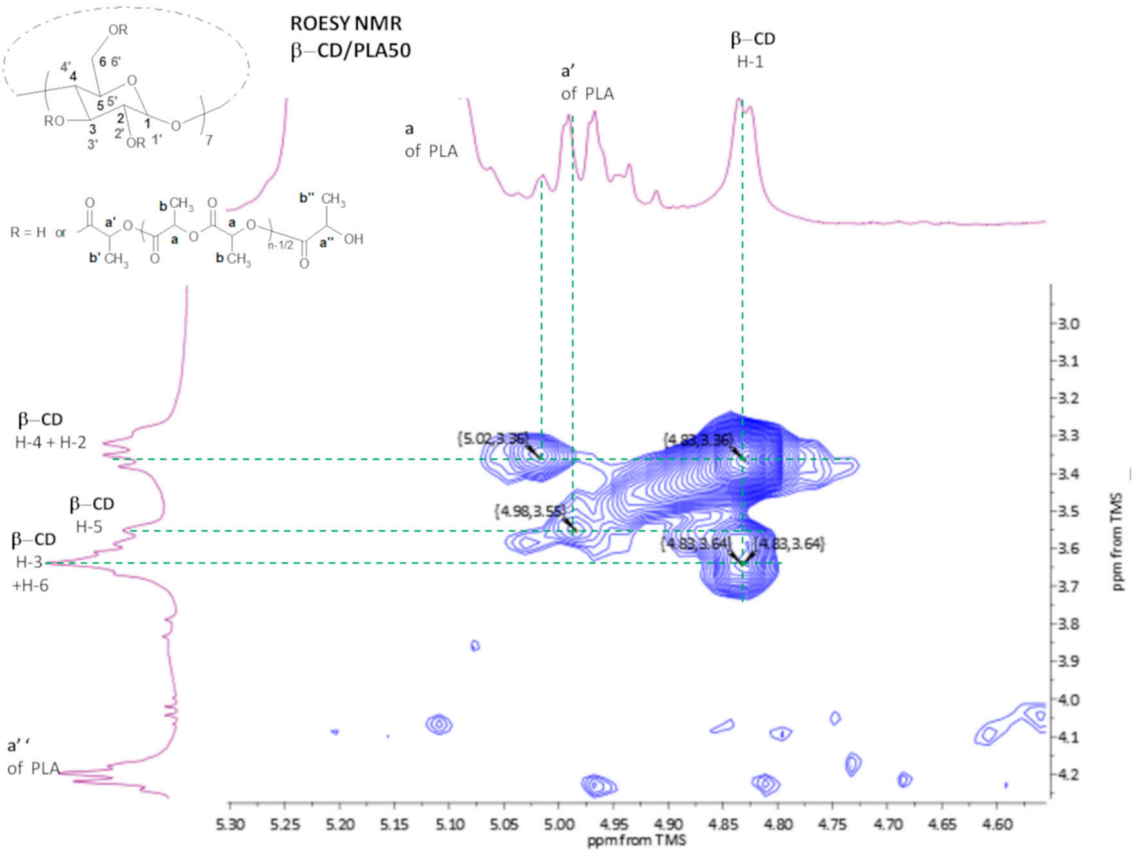

Figure S4. Selected region of ROESY spectrum of the β-CD/PLA50 polymer in DMSO-d$_6$, the plot shows ROE cross-peaks between protons inside the glucose unit of β-CD, and between H-2 and H-5 protons of β-CD and the first mer unit of PLA (signal a').

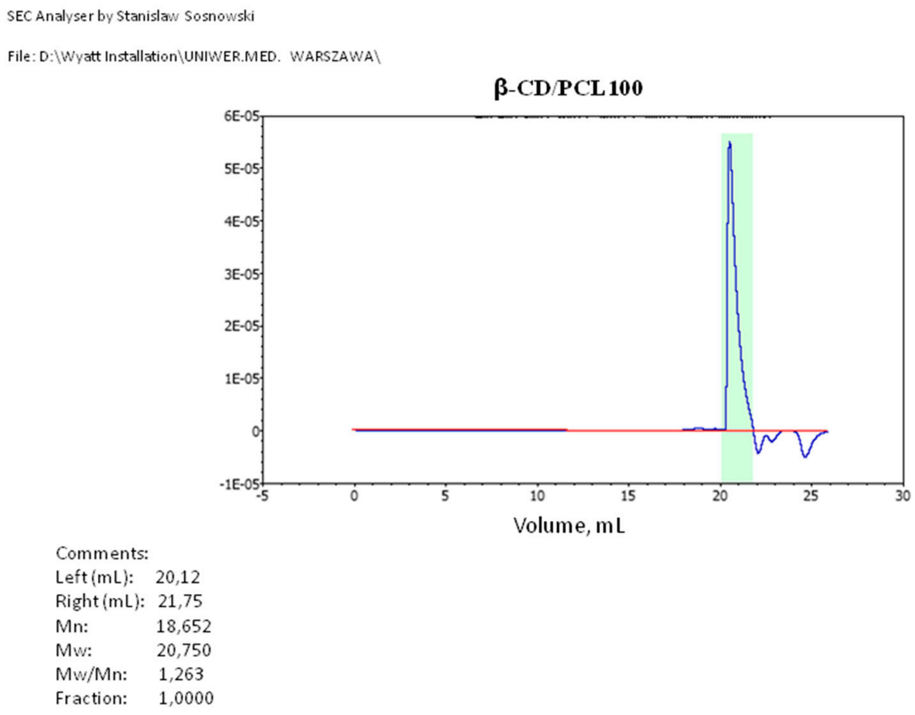

Figure S5. The SEC elution curve of the β-CD/PCL100 polymer sample.

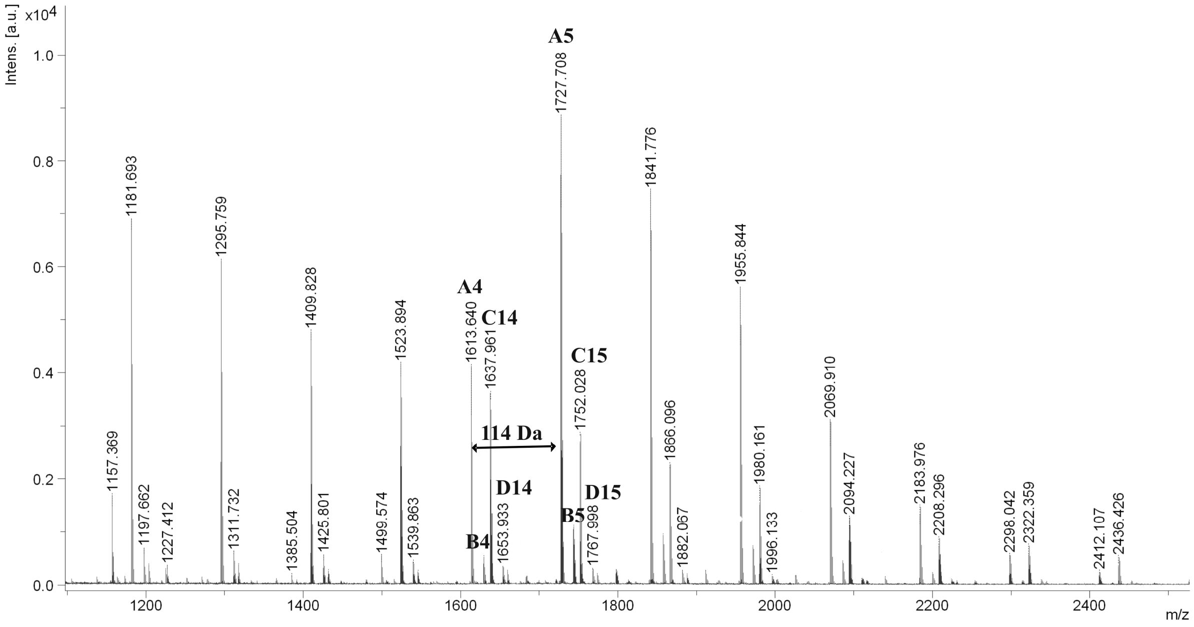

Figure S6. MALDI-TOF MS spectrum of the β-CD/PCL100 polymer obtained at 140 °C.

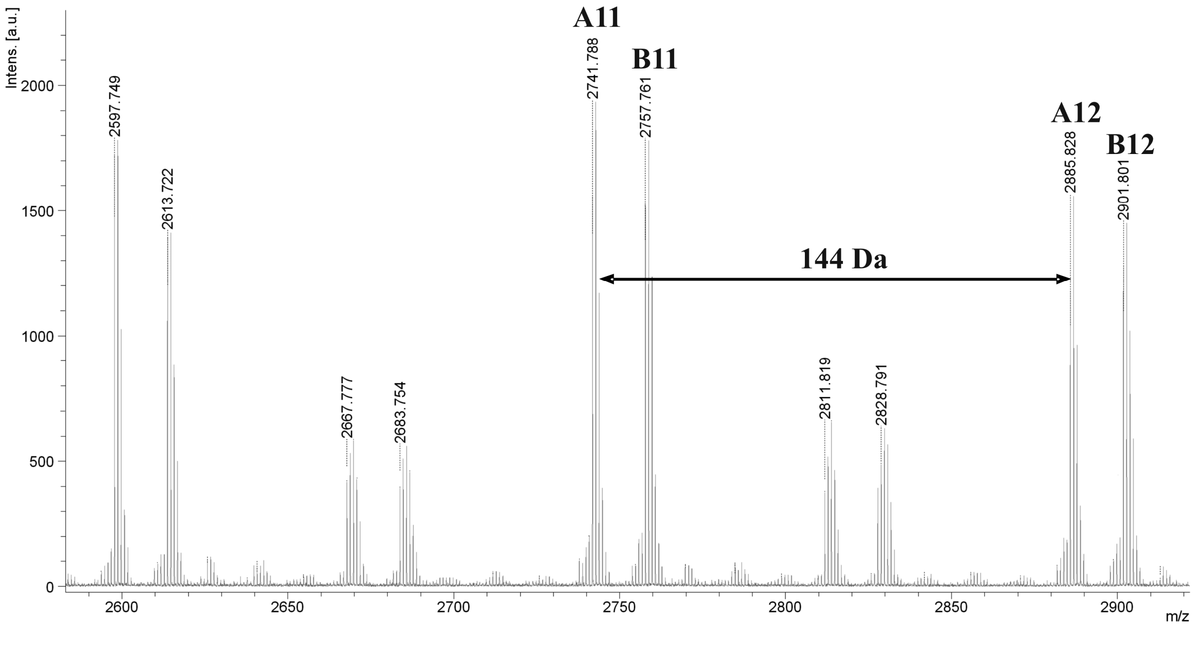

Figure S7. MALDI-TOF MS spectrum of the β-CD/PLA100 polymer obtained at 140 °C.

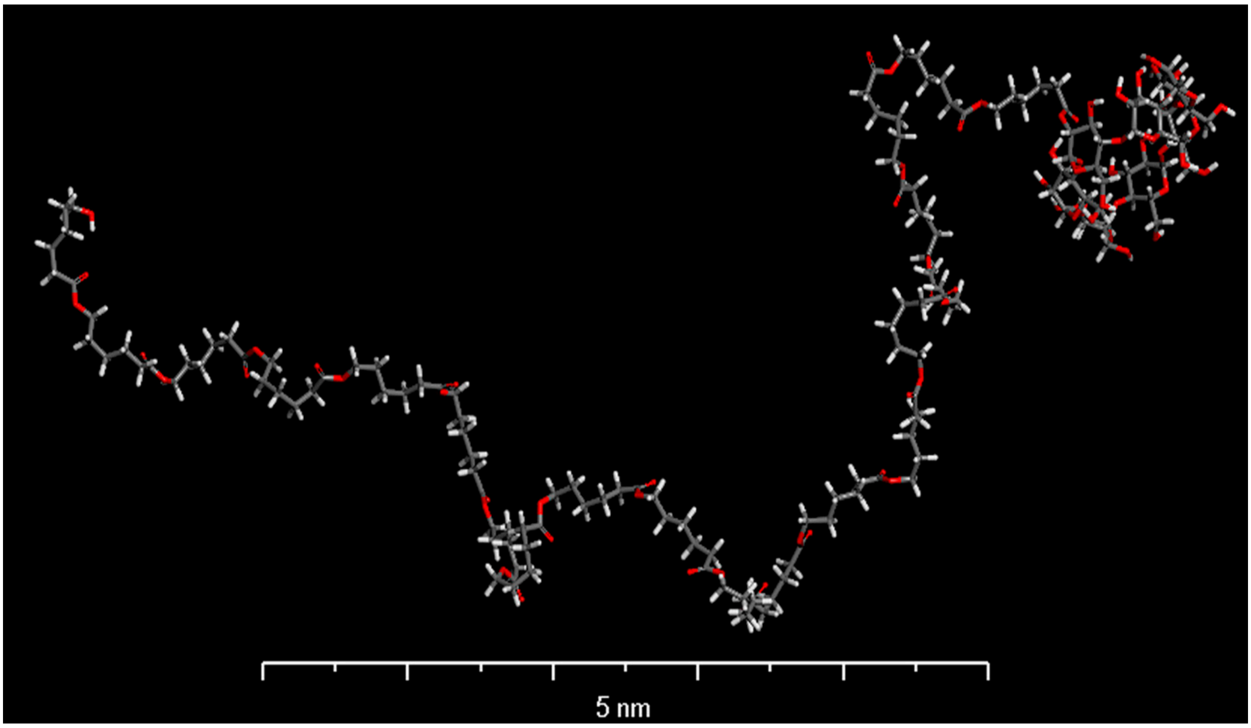

Figure S8. Monosubstituted (at the OH-2 position) β-CD/PCL, DP = 20, DS = 1.

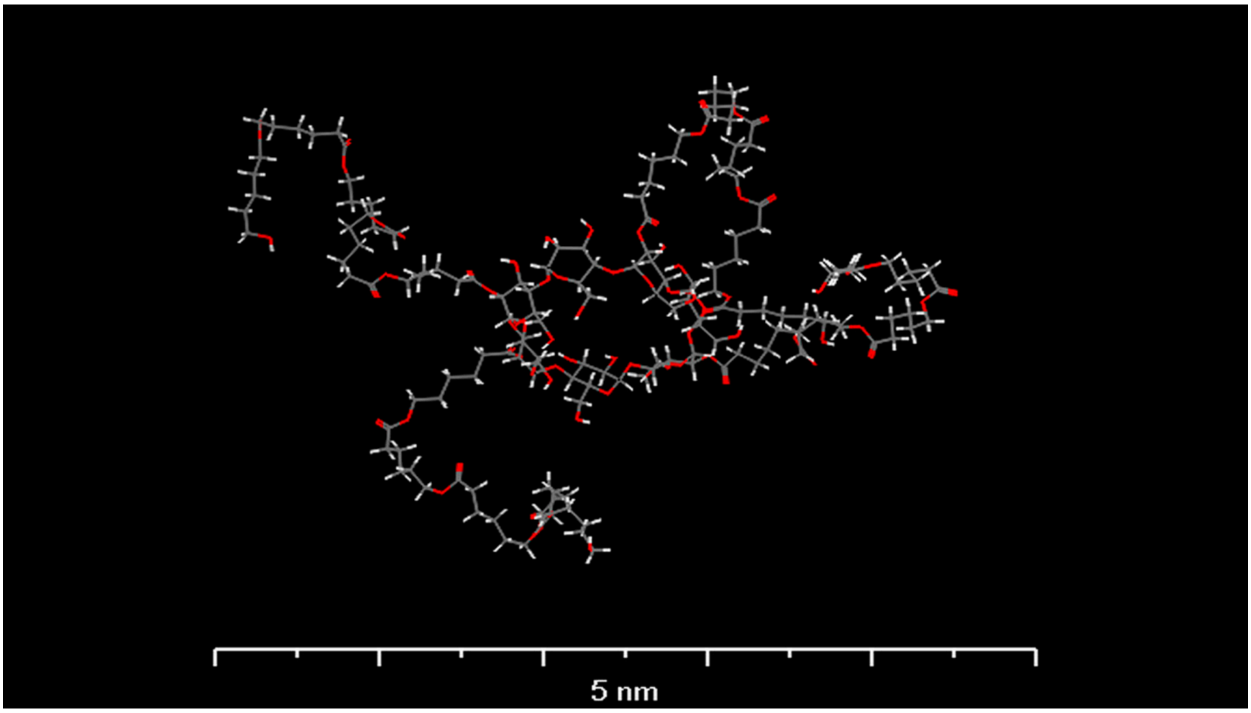

Figure S9. Monosubstituted (at the OH-2 position) β-CD/PCL, DP = 5, DS = 4.

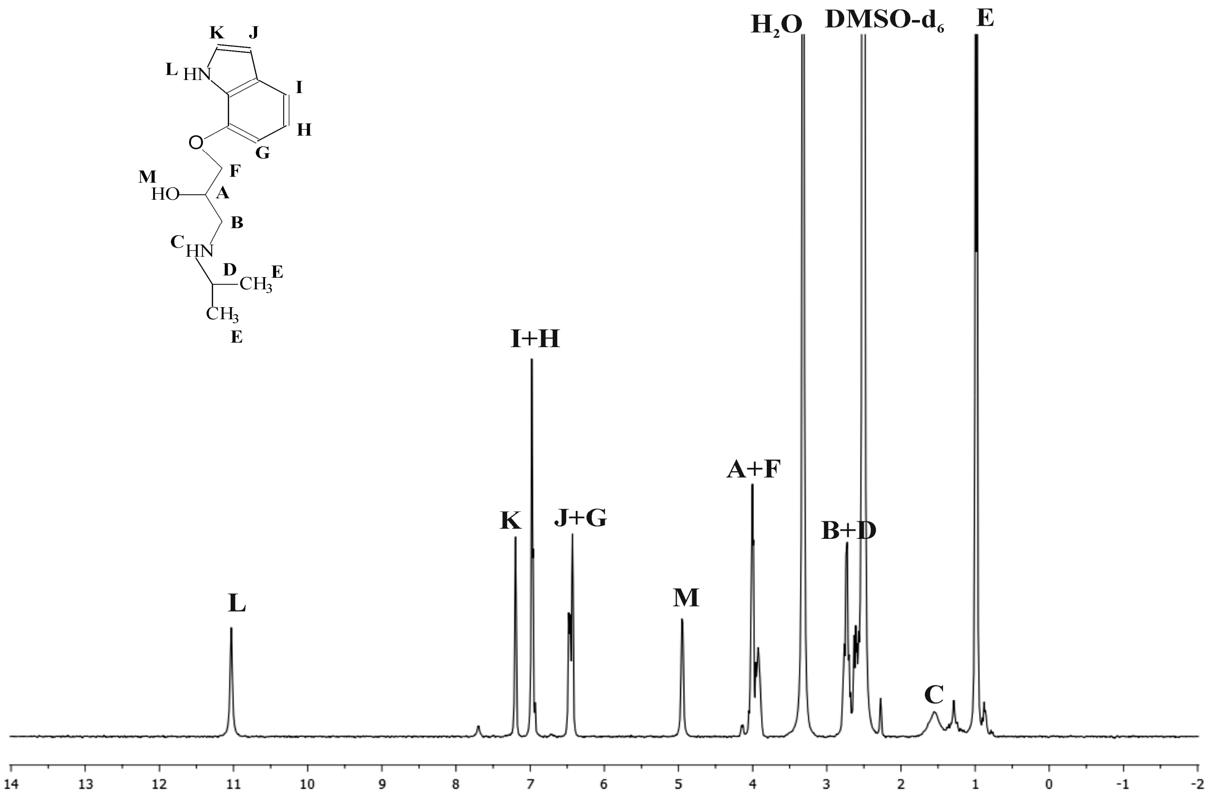

Figure S10. ^{1}H NMR spectrum of pindolol in DMSO-d$_6$.

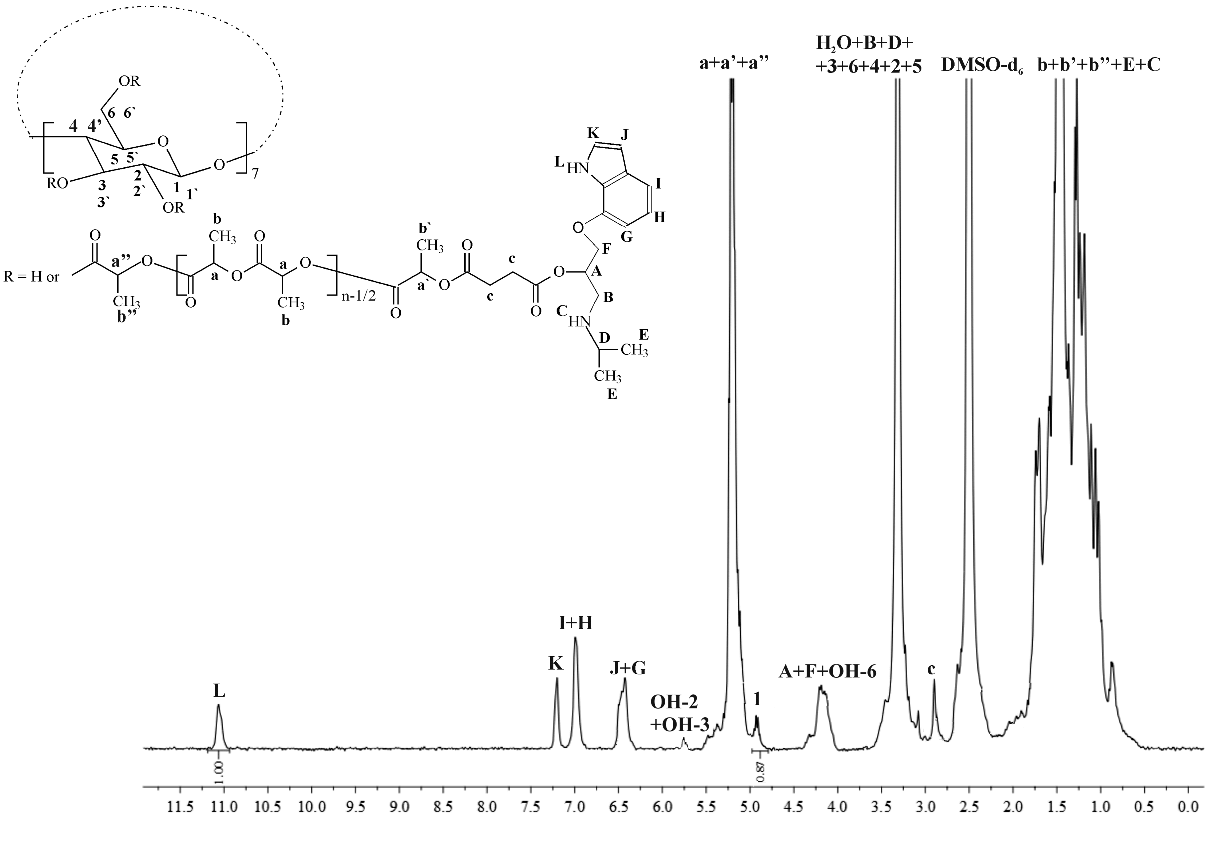

Figure S11. ^{1}H NMR spectrum of the β-CD/PLA100/PDL conjugate in DMSO-d$_6$.
